# RY-2f, an isoflavone analog, overcomes cisplatin resistance to inhibit ovarian tumorigenesis via targeting the PI3K/AKT/mTOR signaling pathway

**DOI:** 10.18632/oncotarget.4634

**Published:** 2015-07-30

**Authors:** Mingming Liu, Zihao Qi, Bingzhi Liu, Yi Ren, Hanbin Li, Gong Yang, Qian Zhang

**Affiliations:** ^1^ Cancer Institute, Fudan University Shanghai Cancer Center; and Department of Oncology, Shanghai Medical College, Fudan University, Shanghai 200032, China; ^2^ Department of Medicinal Chemistry, School of Pharmacy, Fudan University, Shanghai 201203, China; ^3^ Central Laboratory, The Fifth People's Hospital of Shanghai, Fudan University, Shanghai 200240, China

**Keywords:** ovarian cancer, anti-cancer agent, isoflavone analog, PI3K/AKT inhibition, cytotoxicity

## Abstract

Ovarian cancer remains the leading cause of death in gynecologic malignancies partially because of resistance to chemotherapy. In the present study, we show that RY-2f, a chemically synthesized isoflavone analog, inhibited ovarian cancer cell proliferation, blocked cell cycle in G2/M phase and induced cellular apoptosis through up-regulation of p21, cyclin B1, Bax, Bad and cleaved-PARP, and suppression of cyclin A, CDK2 and Bcl-2. We also show that RY-2f could increase the chemotherapeutic efficacy of cisplatin as tested by cell proliferation and colony formation assays, indicating a synergistic effect of RY-2f and cisplatin. Mechanistic study revealed that RY-2f exerted the anti-tumor activities mainly through suppression of the PI3K/AKT/mTOR signaling. Finally, *in vivo* studies showed that RY-2f blocked the A2780-induced xenograft tumor growth without detectable toxicity in the animals at the therapeutic doses, and whereas RY-2f re-sensitized the cisplatin resistant cell line A2780/CDDP induced xenograft tumor to cisplatin treatment. Thus, RY-2f may be developed as a potential therapeutic agent to treat ovarian cancer.

## INTRODUCTION

Ovarian cancer accounts for only about 3% of all malignancies in women, but is the leading cause of death in female reproductive carcinomas [1]. Because most of ovarian cancer cases are diagnosed at advanced stages (60–70%), the 5 year survival rate is around 46% [2]. The current standard treatment for ovarian cancer includes radical surgery and platinum- or taxane-based chemotherapy [3, 4]. Despite the initial response to standard treatment, majority of advanced ovarian cancers recur at a median of 18–24 months after diagnosis [5]. However, drug-resistance has been a major challenge in treatment of recurrent ovarian cancer [6]. Therefore, it is urgent to develop novel therapeutic agents for relapsing ovarian cancer patients, especially for those resistant to platinum-based chemotherapy.

Numerous investigations have demonstrated that the PI3K/AKT/mTOR signaling is commonly over-activated and plays an important role in the stimulation of proliferation, survival, metastasis, and drug-resistance in many cancer types [7–10]. Particularly, amplification or mutations of *PIK3CA* (encoding the catalytic subunit of PI3K P110α), loss of PTEN, and deregulation of AKT are well-known mechanisms that activate this pathway in approximately 70% of ovarian cancers [11–15]. In addition, studies have also shown that the activation of the PI3K/AKT/mTOR signal pathway contributes to the platinum-based resistance and poor prognoses in ovarian cancer [16–18]. Therefore, to improve the sensitivity of ovarian cancer cells to platinum-based chemotherapy, targeting the PI3K/ATK/mTOR signal pathway has emerged as one of the major therapeutic strategies [19, 20].

Isoflavones (molecules containing 3-phenyl-4H-chromen-4-one) enriched in soy beans and soy germ have been reported to possess chemo-preventive and chemotherapeutic potentials in both hormone-and non-hormone-dependent tumor types, including ovarian, prostate, breast, colon, gastric, lung, and pancreatic tumors [21–23]. Studies from cancer epidemiology revealed that the intake of soy and isoflavones has a negative association with ovarian cancer risk [24–26]. Another study showed that the incidence of ovarian cancer is much lower in Japan than in Western countries, because Japanese women consume large amounts of isoflavone-rich soy foods in their dietary [27]. Additional investigations have shown that isoflavones, such as genistein, glycitein, and daidzein, exert *in vitro* and *in vivo* pleiotropic anti-tumor effects through suppression of cell cycle, induction of apoptosis, inhibition of angiogenesis and metastasis, and anti-oxidation [22, 28–30]. Hence, isoflavones have been recognized as promising candidates in the development of anti-tumor agents.

**Figure 1:**
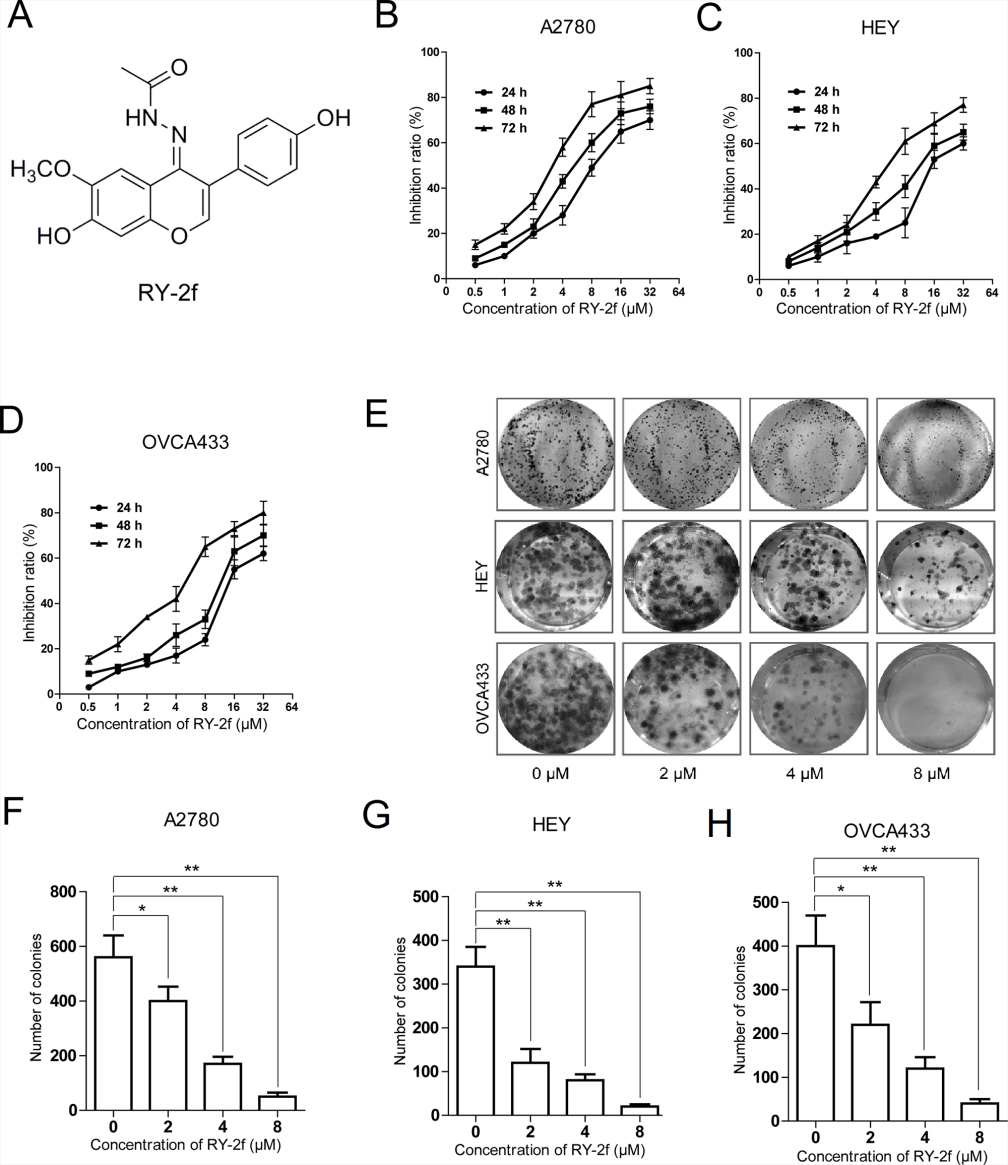
RY-2f inhibits cell proliferation and colony formation **A.** Chemical structure of the isofalvone analog, RY-2f. **B-D.** RY-2f inhibits the proliferation of ovarian cancer cell lines A2780 (B), HEY (C) and OVCA433 (D). Cell viability determined by MTT assay. **E.** Representative images of cell colonies after treatment with various concentrations of RY-2f for 48 h. **F-H.** Colony formation rate after treatment with RY-2f for 48 h. The experiments were repeated three times, and a representative experiment is shown. *p < 0.05, **p < 0.01.

From a series of synthesized isoflavone analogs, we identified a novel isoflavone analog, (*Z*)-*N’*-(7-hydroxy-3-(4-hydroxyphenyl)-6-methoxy-4H-chromen-4-ylidene) acetohydrazide, named as RY-2f (Figure 1A), and herein, we report its *in vitro and in vivo* anti-tumor functions.

## RESULTS

### RY-2f suppresses cell proliferation

The anti-proliferative activity of RY-2f was initially tested by using human ovarian carcinoma cells, including A2780, HEY and OVCA433 cell lines. Cells were treated with different concentrations of RY-2f for 24, 48, and 72 hours, and the cell viability was determined by MTT assay. As shown in Figure 1B, treatment of A2780 cells with RY-2f resulted in a corresponding decrease of cell proliferation and viability in a dose- and time-dependent manner. Similar effects were obtained on treatment of HEY (Figure 1C) and OVCA433 (Figure 1D) cells. The IC50 values were calculated and listed in Table 1. In contrast, the sensitivity of normal human ovarian epithelial cells (T29) [31] to RY-2f was much low (Table 1), suggesting that RY-2f has selective cytotoxicity on ovarian cancer cells, but possesses less cytotoxicity on normal ovarian epithelial cells. Moreover, we also tested the anti-proliferative activities of glycitein (4′, 7-Dihydroxy-6-methoxyisoflavone), which is the leading compound of RY-2f, and another isoflavone compound, genistein (4′, 5, 7-trihydroxyisoflavone). As listed in Table 1, compared with RY-2f, glycitein and genistein exhibited weaker effect on the inhibition of both neoplastic and pre-neoplastic cell growth, which may indicate that the nitrogen-containing groups are essential for the anti-cancer activities.

**Table 1:** The IC_50_ values of RY-2f, glycitein and genistein against ovarian cancer cells and normal ovarian epithelium cells

Cell lines	IC_50_								
	RY-2f			glycitein			genistein		
	24 h	48 h	72 h	24 h	48 h	72 h	24 h	48 h	72 h
A2780	6.7	4.8	2.3	> 64	40.4	19.8	45.3	30.7	15.8
HEY	10.3	7.7	4.2	> 64	> 64	45.9	> 64	32.4	23.6
OVCA433	11.2	8.5	5.3	> 64	> 64	34.1	49.0	28.7	14.1
Normal ovarian epithelium cells (T29)	> 64	> 64	> 64	> 64	> 64	> 64	> 64	> 64	> 64

Next, we employed colony formation assay to further confirm the cytotoxicity of RY-2f. Colony formation assay is based on the ability of a single cell to proliferate and to form a colony. Thus, it has been used to determine the cytotoxicity induced by various chemotherapeutic agents [32]. In the present study, as shown in Figure 1E, treatment of A2780, HEY and OVCA433 cells with RY-2f at the different concentrations (2, 4 and 8 μM) for 48 h dose-dependently reduced the number of colonies, compared with cells treated with diluent (DMSO). The numbers of colonies formed by cells treated with RY-2f or diluent were shown in Figure 1F-H.

### RY-2f induces cell cycle arrest

**Figure 2:**
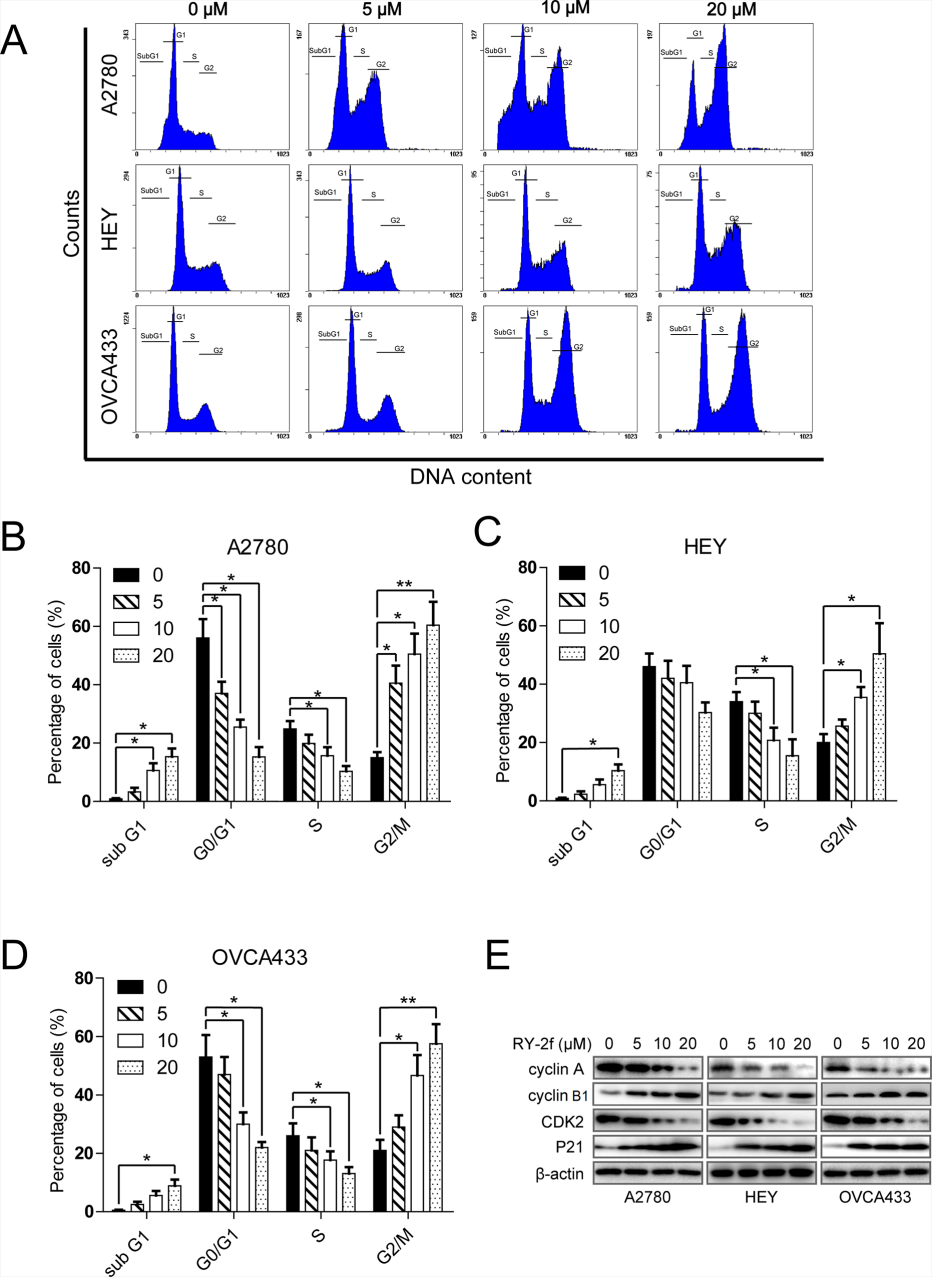
RY-2f suppresses cell cycle progression **A.** Cell cycle distribution after treatment with different concentrations of RY-2f for 24 h. **B-D.** Quantitative analysis of A2780 (B), HEY (C) and OVCA433 (D) cells treated with RY-2f. **E.** Regulation of cell cycle associated proteins. The experiments were repeated three times, and a representative experiment is shown. *p < 0.05, **p < 0.01.

Anti-tumor chemicals usually inhibit cell proliferation through induction of cell cycle arrest. Therefore, to test how the cell cycle was inhibited by RY-2f, the DNA-based cell cycle was analyzed by flow cytometry. We first treated cells with RY-2f for 24 h and then examined the DNA content after propidium iodide (PI) staining. We found that the cell population was dose-dependently increased in the G2/M phase but decreased in the S phase in all three cell lines treated with the various concentrations of RY-2f, when compared with control cells treated with diluent (Figure 2A). Moreover, the apoptosis induced by RY-2f was observed as the hypodiploid DNA content shown in so-called “sub-G1” peaks in DNA histograms. The cell cycle distribution of the cells treated with RY-2f or diluent were shown in Figure 2B-D. These data indicate that RY-2f induces cell cycle arrest through reducing S phase and accumulating G2/M phase populations in cancer cells.

Further, we examined the effect of RY-2f on cell cycle regulatory molecules including p21^Cip1^, cyclin A, cyclin B1 and CDK2 by Western blot. As shown in Figure 2E, treatment of cells with RY-2f dose-dependently up-regulated p21^Cip1^ and cyclin B1, but down-regulated cyclin A and CDK2 in three cell lines. These data suggest that the G2/M cell cycle arrest caused by RY-2f may be associated with up-regulation of the cell cycle inhibitory proteins and down-regulation of the cell cycle transition-promoting proteins.

### RY-2f induces cell apoptosis through mitochondrial apoptotic pathway

**Figure 3:**
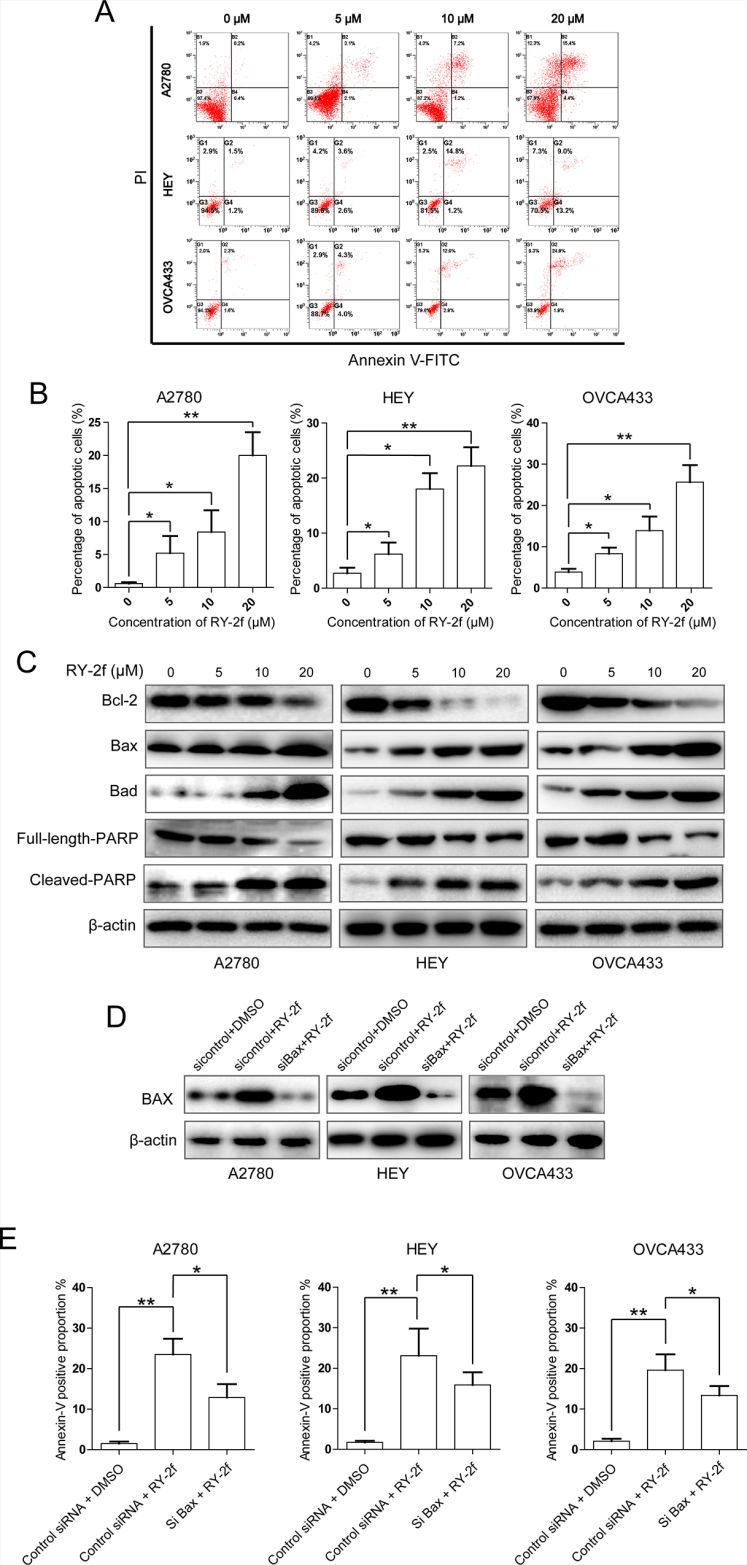
RY-2f induces apoptosis through mitochondrial pathway **A.** Representative flow cytometry profiles of apoptosis. **B.** Quantitative results obtained using Annexin V/PI staining. **C.** Western blot analysis of apoptosis-related proteins. **D.** Silencing of Bax by specific siRNA. **E.** Knockdown Bax significantly attenuates RY-2f-induced apoptosis in the three ovarian cancer cell lines.

To examine cellular apoptosis, cells double-stained with Annexin-V/PI were subjected to the flow cytometry analysis. We first treated cells with RY-2f at the concentrations of 5, 10 and 20 μM for 24 h, and then stained the cells with Annexin-V and PI. We found that the total proportion of Annexin V positive cells, including Annexin V^+^/PI^−^ (the right lower quadrant, representing early apoptosis) and Annexin V^+^/PI^+^ (the right upper quadrant, representing late apoptosis and necrosis) cells, were dose-dependently increased with the raising concentrations of RY-2f in all three ovarian cancer cell lines (Figure 3A and 3B).

To gain insight into the mechanism of apoptosis induced by RY-2f, we subsequently examined apoptosis-associated proteins by immunoblotting analysis of cell lysates with corresponding antibodies. As indicated in Figure 3C, compared with in control cells, RY-2f dose-dependently down-regulated Bcl-2, but up-regulated Bax and Bad in three cell lines after treatment for 24 h, while β-actin served as a loading control. Moreover, compared with control cells, the exposure of cells to RY-2f promoted the cleavage of poly(ADP-ribose) polymerase-1 (PARP-1) in a dose-dependent manner, which is a marker of cells undergoing apoptosis. In order to investigate whether the RY-2f-induced apoptosis is mediated through the mitochondrial apoptotic pathway, Bax was silenced by a specific siRNA in three cell lines (Figure 3D). The apoptosis induced by RY-2f were significantly attenuated by knockdown of Bax in all three cell lines, compared with control cells (Figure 3E). These results indicate that RY-2f triggers mitochondrial dependent apoptosis in ovarian cancer cells.

### RY-2f sensitizes cancer cells to cisplatin treatment

**Figure 4:**
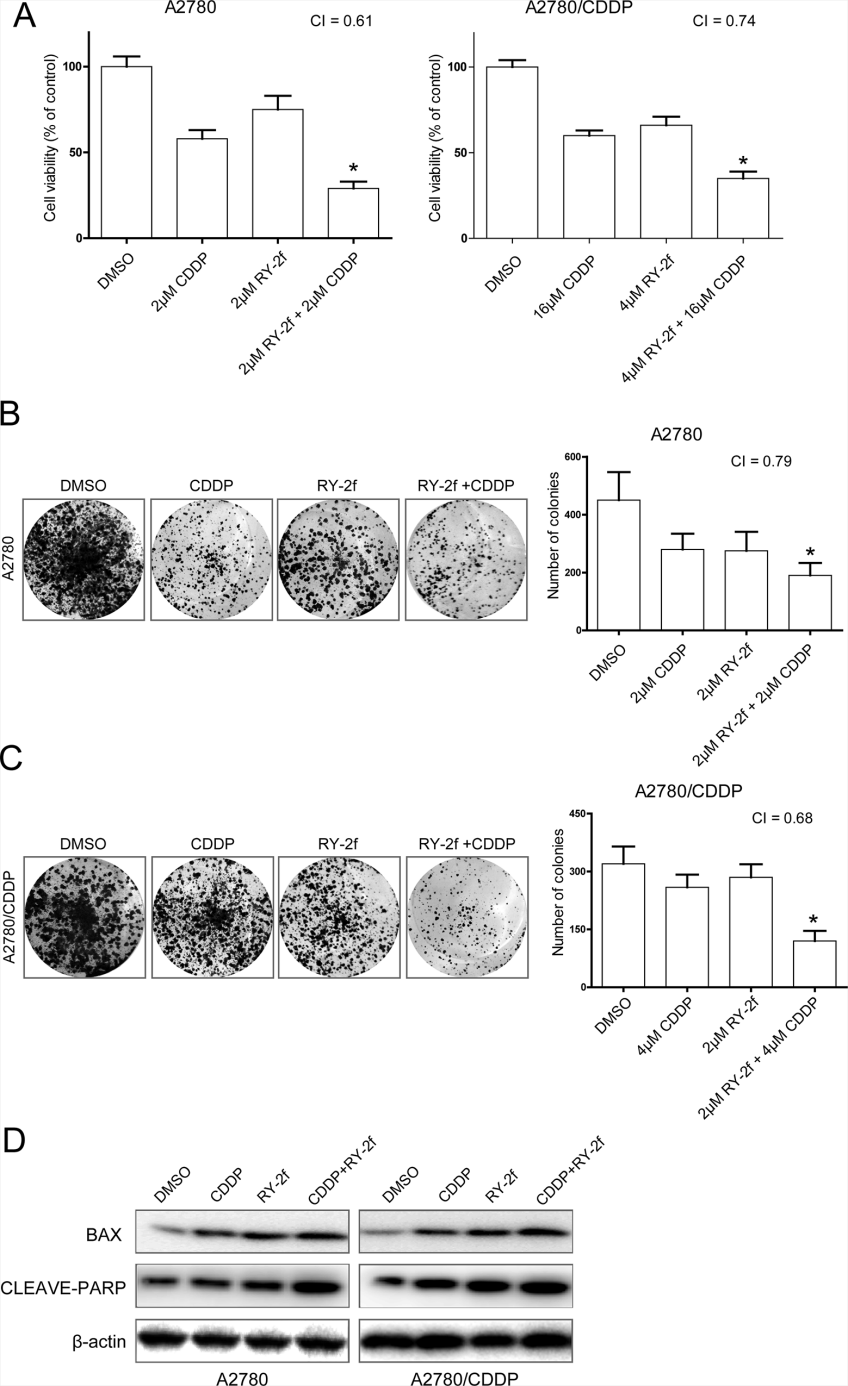
RY-2f sensitizes cancer cells to cisplatin treatment **A.** The concurrent administration of cells with RY-2f and cisplatin for 48 h results in synergistic inhibitory effect on the growth of A2780 and A2780/CDDP cells. **B-C.** Representative images and number of colonies formed by A2780 (B) or A2780/CDDP (C) Cells treated with cisplatin or RY-2f alone, and cisplatin plus RY-2f. The experiments were repeated three times, and a representative experiment is shown. *p < 0.05, **p < 0.01. **D.** Western blot analysis showing that combination of RY-2f and cisplatin induces more expression of Bax and cleavage of PARP than each agent alone.

Since the activation of AKT is shown to contribute to the resistance of platinum therapy in ovarian cancer [16, 33], we next tested if RY-2f could improve sensitization of ovarian cancer cells to cisplatin treatment. We treated A2780 and A2780 cisplatin resistant cells (A2780/CDDP) with cisplatin alone or cisplatin plus RY-2f. As shown in Figure 4A, treatment of cells with cisplatin alone had a fairly low anti-proliferative effect, especially on A2780/CDDP cells. However, the combined administration of cells with RY-2f and cisplatin resulted in highly reduced cell growth at 48 h compared with cisplatin alone. The combination index (CI) was 0.61 and 0.74, indicating a synergistic effect (CI < 1) of cisplatin and RY-2f. We then confirmed the synergistic function of RY-2f in cisplatin-treated cells by colony formation assay. Concurrent treatment of A2780 or A2780/CDDP cell lines with both cisplatin and RY-2f resulted in greater inhibition of colony formation than did treatment of cells with either agent alone (Figure 4B and 4C). In addition, the combined treatment with cisplatin and RY-2f promoted more up-regulation of Bax and more cleavage of PARP-1 than the treatment with either agent alone in three cell lines, suggesting a strong synergistic induction of apoptosis by both agents (Figure 4D).

### RY-2f disrupts the PI3K/AKT/mTOR signal pathway

**Figure 5:**
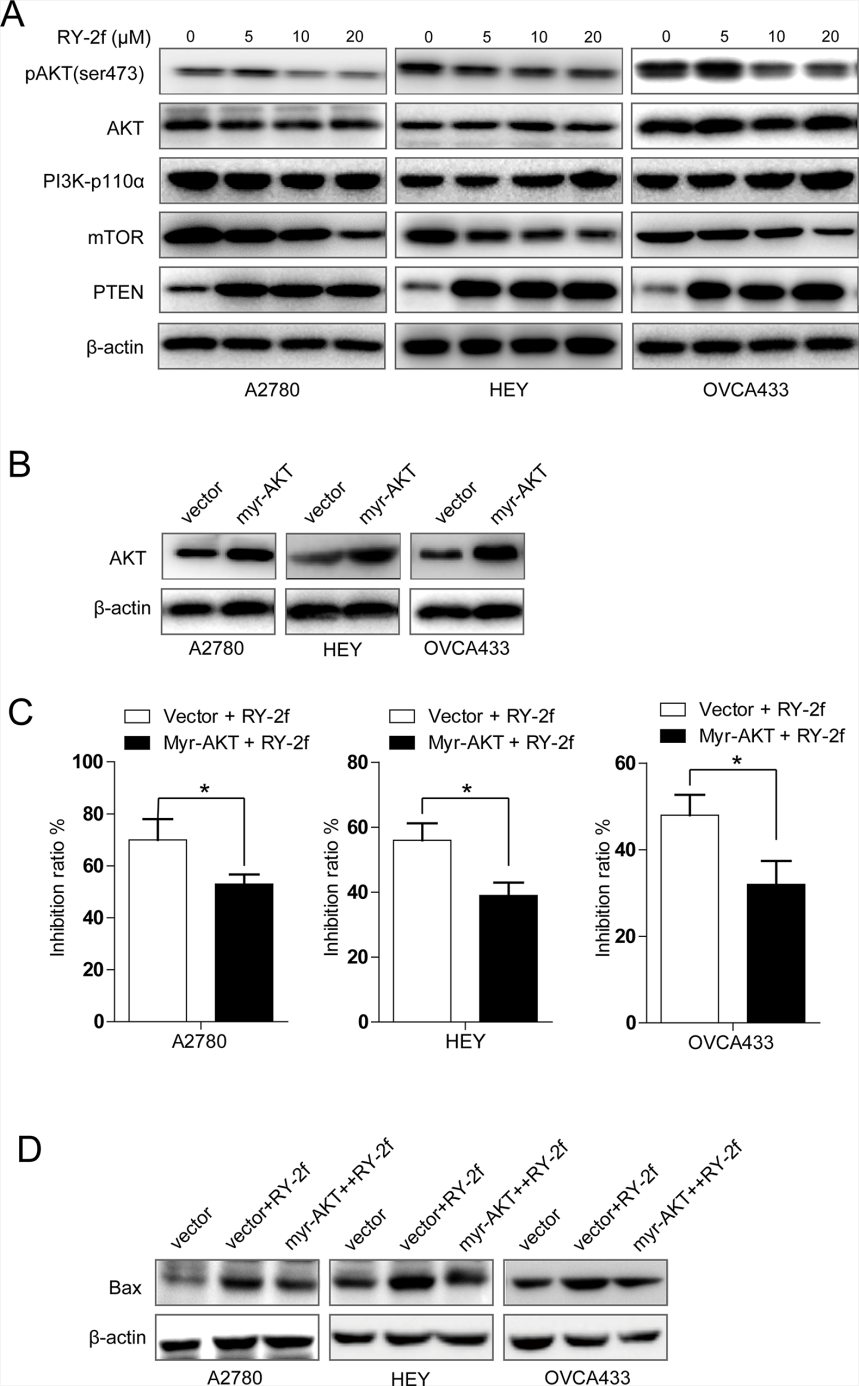
RY-2f suppressed PI3K/AKT/mTOR signaling pathway **A.** Suppression of phosphorylated AKT^Ser473^ and mTOR and induction of PTEN by RY-2f in A2780, HEY, and OVCA433 cells. β-Actin was used as an equal loading control. **B.** Analysis of cells transfected with AKT1 constitutively active plasmid or control vector. **C.** Introduction of constitutively active AKT1 significant blocks the anti-proliferative activity of RY-2f. **D.** Constitutively active AKT1 inhibited the RY-2f induced up-regulation of Bax. The assay was repeated three times, and a representative result is shown. *p < 0.05, **p < 0.01.

Several natural compounds or their synthetic analogs, such as isoflavones and bisflavones, have been demonstrated to overcome cisplatin resistance in ovarian carcinoma through suppression of the PI3K/AKT/mTOR signaling [33–35]. Therefore, we employed Western blot to test whether the PI3K/AKT/mTOR signal pathway was involved in the RY-2f-mediated anti-cancer effects. Indeed, as shown in Figure 5A, with the increase of RY-2f concentration, the phosphorylation of AKT at Ser473 as well as the expression of mTOR, a known downstream target of AKT, were dose-dependently suppressed in A2780, HEY and OVCA433 cells, whereas no changes of PI3K p110 α and total AKT were observed. Moreover, RY-2f up-regulated the expression of PTEN, a key negative regulator of the PI3K/AKT pathway, in all three cell lines.

To further confirm above notion in RY-2f mediated anti-cancer effects, we transfected A2780, HEY and OVCA433 cells with a plasmid containing constitutively active AKT1 (myr-AKT1) or control vector, respectively (Figure 5B), and then determined the anti-proliferative effect of RY-2f on the transfected cells. As shown in Figure 5C, introduction of myr-AKT1 significantly rescued the anti-proliferative effect of RY-2f. In addition, we also determined the expression of Bax in cells transfected with myr-AKT plasmid to explore the association between AKT phosphorylation and apoptosis-related protein. As shown in Figure 5D, activation of AKT led to the reduction of RY-2f-induced up-regulation of Bax in myr-AKT1 transfected cells, compared with that in vector-transfected control cells (Figure 5D). Collectively, these data suggest that RY-2f exerts its anti-cancer function, at least partially, through disruption of the PI3K/AKT/mTOR signal pathway, which may consequently result in cellular apoptosis.

### RY-2f suppresses xenograft ovarian tumor growth

**Figure 6:**
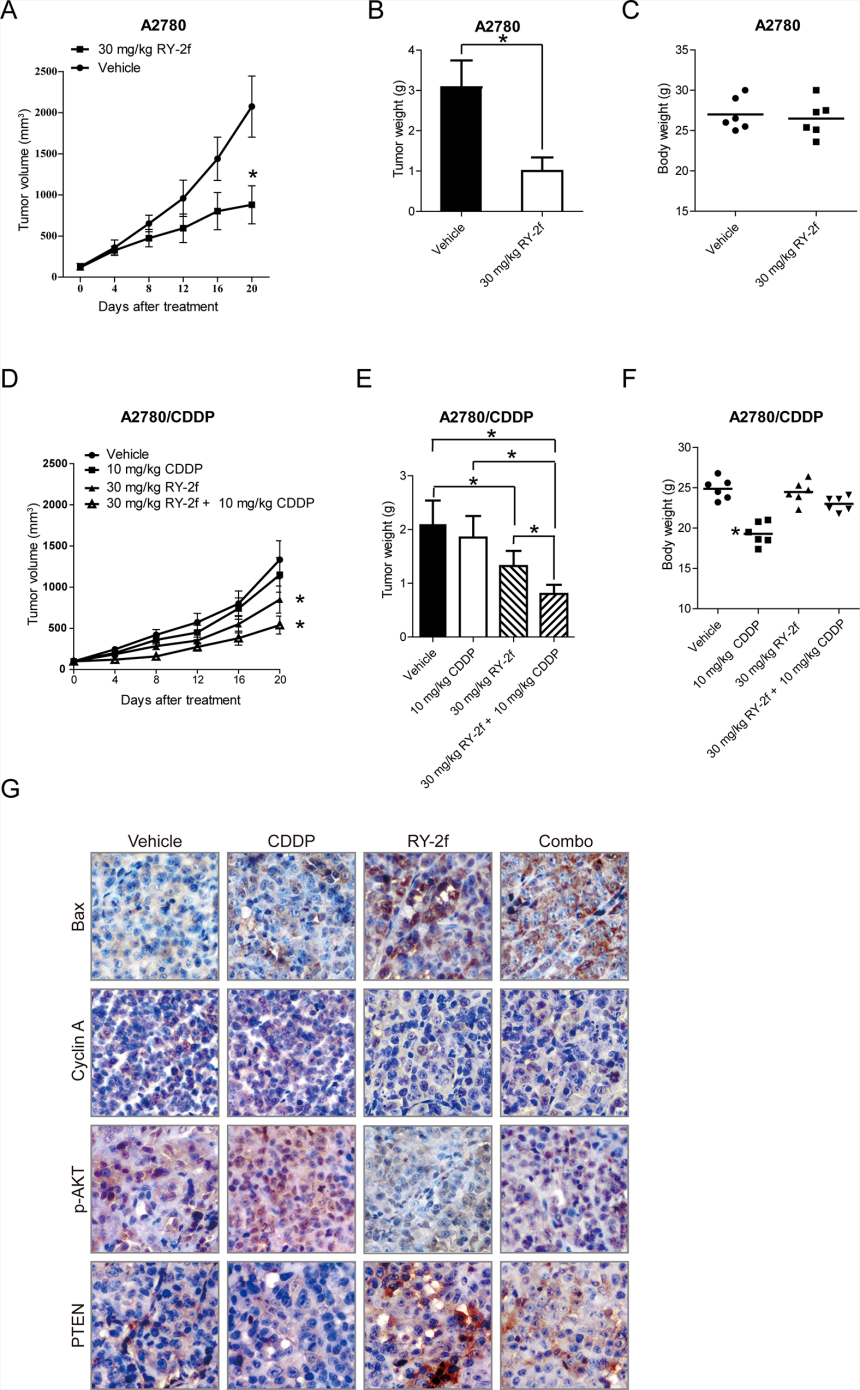
Inhibition of xenograft ovarian cancer by RY-2f **A-C.** Nude mice bearing tumors formed by A2780 cells were administered with 30 mg/kg of RY-2f, or vehicle as controls (*n* = 6). Treatment was done given through intraperitoneal injection of RY-2f once every four days. Figures show the average tumor volumes (A), weights (B), and body weights of mice at the end of observation (C). **D-E.** Growth-inhibition of xenograft tumor generated with A2780/CDDP cells by combined treatment with CDDP (10 mg/kg, i.p. per 4 days) and RY-2f (30 mg/kg, i.p. per 4 days). Compared to CDDP alone or vehicle treatment, the combination treatment resulted in a significant reduction in tumor growth (D) and average tumor weight (E). Compared to vehicle treatment, CDDP treatment reduced the body weights of animals, while the combined treatment with CDDP and RY-2f slightly reduced body weights, but did not reach statistical significance **F. G.** Representative images showing expression levels of Bax, Cyclin A, p-AKT, and pTEN detected by immunostaining of xenograft tumor tissues from animals with different treatments.

To assess the *in vivo* anti-tumor activity of RY-2f, xenograft tumors were established by subcutaneous inoculation of A2780 cells in female BALB/C nude mice. After solid tumor reached to 100 mm^3^, animals were randomly divided into two groups and administrated with either 30 mg/kg of RY-2f or vehicle once every four days. As shown in Figure 6A, compared with mice treated with vehicle, animals treated with RY-2f appeared with 55.8% reduction of tumor growth after 6 administrations (20 days). The average tumor weight from diluent-treated mice was more than that of RY-2f-treated animals (Figure 6B). However, no any significant difference of body weights between RY-2f-treated and diluent-treated mice was observed (Figure 6C), indicating that RY-2f may own the property of low toxicity on tested animals.

Furthermore, we tested the growth-inhibitory effect on xenograft animals generated with A2780/CDDP by CDDP (10 mg/kg, i.p. per 4 days) or RY-2f (30 mg/kg, i.p. per 4 days) as single agents, or by the combination of CDDP (10 mg/kg, i.p. per 4 days) and RY-2f (30 mg/kg, i.p. per 4 days). The results showed that CDDP alone was not significantly effective on reduction of tumor volume. In comparison, RY-2f treatment and the combinative treatment resulted in a significant reduction of tumor growth (Figure 6D). Correspondingly, the tumor weight from RY-2f and combinative treatment was significantly decreased, compared with diluent or CDDP treated group (Figure 6E). Besides, compared with vehicle, CDDP treatment reduced body weight of mice, while the RY-2f and combined treatment slightly reduced body weight, but did not reach statistical significance (Figure 6F). Furthermore, we performed the immunohistochemistry assay to determine the expression of p-AKT, PTEN, cyclin A, and Bax in the xenograft tumor tissues. As shown in Figure 6G, CDDP treatment up-regulated the phosphorylation of AKT and cyclin A, while consistent with the *in vitro* results, RY-2f treatment decreased the phosphorylation of AKT and cyclin A in tumor tissues. The combinative administration further up-regulated the pro-apoptotic factor Bax. These *in vivo* findings demonstrate that RY-2f enhances the anticancer efficacy of cisplatin treatment in platinum resistant ovarian cancer cells.

## DISCUSSION

Naturally occurring products and their synthetic analogs have always been important sources for discovering new therapeutic agents [36, 37]. In the present study, as a part of our efforts to develop novel chemotherapeutic agents, the isoflavone analog RY-2f was identified with strong inhibition of ovarian cancer cell proliferation. Importantly, in agreement with the *in vitro* data, RY-2f also displays the activity to suppress *in vivo* A2780 xenograft tumor growth with little toxicity in tested animals at the therapeutic dose (Figure 6A-C).

Dysregulation of cell cycle is one of the major characteristics in many cancers. Naturally derived isoflavone compounds have exhibited their anti-tumor activities through induction of cell cycle arrest in different human cancer cell types [29]. The cyclin A/CDK2 complex is responsible for cell cycle progression in late S phase and early G2 phase and decreases in late G2 phase and mitosis [38]. p21, which belongs to Cip/Kip family, negatively regulates cell cycle progression through inhibition of CDK-cyclin complexes [39, 40]. In this study, we show that RY-2f induces G2/M cell cycle arrest with down-regulation of cyclin A and CDK2, and up-regulation of p21 (Figure 2C), indicating that cancer cells may exit from G2 phase and enter M phase in response to RY-2f treatment. Cyclin B1, which is synthesized at interphase and reaches maximum level at metaphase, has been identified to regulate the progression from late G2 phase to mitosis as complex with CDK1 [41]. To progress from metaphase to anaphase, cyclin B1 is degraded rapidly by the anaphase-promoting complex [42]. Our results showed that RY-2f significantly up-regulated the accumulation of cyclin B1, which may indicate that the accumulation of cyclin B1 finally induced the cell cycle arrest and programmed cell death [43].

Apoptosis, a major form of cell death, is regulated by numerous molecular signaling pathways including mitochondrial pathways. In this pathway, mitochondrial outer membrane permeabilization is tightly controlled by the interaction of anti- and pro-apoptotic proteins of Bcl-2 family [44]. The ratio between anti- and pro-apoptotic Bcl-2 family proteins has been recognized as an effective marker to judge whether a cell will go to apoptosis [45]. Our investigation showed that treatment of cells with RY-2f resulted in increase of the pro-apoptotic proteins Bax and Bad, but led to decrease of the anti-apoptotic protein Bcl-2 (Figure 3C). Knockdown of Bax by specific siRNA significantly suppressed the RY-2f-induced apoptosis (Figure 3E). Therefore, our findings indicate that the apoptosis induced by RY-2f is dependent on activation of the mitochondrial apoptotic pathway.

Cisplatin is one of the first-line chemotherapeutic agents widely used to treat various cancers. However, resistance to cisplatin has been a major clinical obstacle in ovarian cancer therapy [46, 47]. Our data suggest that RY-2f sensitizes both cisplatin sensitive and resistant ovarian cancer cell lines A2780 and A2780/CDDP to cisplatin treatment (Figure 4 A-C). Moreover, we also show that RY-2f is able to overcome the resistance of cisplatin in xenograft tumor models (Figure 6D and E).

The PI3K/AKT/mTOR pathway, serving as a proto-oncogenic pathway, has been critical for cancer progression, including cellular proliferation, growth, survival, and drug resistance [48]. Over-activation of the signaling pathway has been observed in a variety of tumors including ovarian cancer, multiple myeloma, breast cancer, prostate cancer, and so on [49, 50]. Once a cell signal is received from external growth factors, the corresponding receptors, such as the epidermal growth factor receptor (EGFR), can be dimerized to trigger the AKT signaling cascade through PI3K, which subsequently activates transcription factors like mTOR and NF-κB to stimulate the transcription of pro-survival genes [51]. Meanwhile, mTORC2 may be activated by insulin-stimulated PI3K. Once activated, mTORC2 phosphorylates Ser473 of AKT to activate AKT [52]. In addition, studies have also revealed that the activation of AKT can suppress the pro-apoptotic factor Bad to inhibit apoptosis and counteract the function of p21 to promote cell cycle progression [53–55]. In this study, western blot and immunohistochemistry analysis show that the phosphorylation of AKT at Ser473 were attenuated by RY-2f *in vitro* (Figure 5A) and *in vivo* (Figure 6G). In addition, introduction of cells with constitutively active AKT1 significantly rescued the inhibitory effect of RY-2f (Figure 5C), clearly indicating that the anti-cancer effect is dependent on suppression of the PI3K/AKT /mTOR pathway. Besides, we also found that RY-2f dramatically induced the expression of PTEN (Figure 5A), an important tumor suppressor and negative regulator of the PI3K/AKT pathway, but didn't affected the expression of PI3K and AKT. Collectively, it might be proposed that the primary target of RY-2f is AKT, and RY-2f may directly interact with AKT to inhibit its phosphorylation. However, further investigation is needed to validate this hypothesis through protein kinase assay.

In summary, by *in vitro* and *in vivo* experiments, we have revealed the potential therapeutic function of RY-2f, a synthetic isoflavone analog, using human ovarian cancer cells as a model. RY-2f induces cellular apoptosis and cell cycle arrest, overcomes cisplatin resistance, and inhibits xenograft tumor growth mainly through repression of the PI3K/ATK pathway. Therefore, RY-2f may be developed as a potential drug to treat human ovarian cancer after further validation.

## MATERIALS AND METHODS

### Drugs

RY-2f (Figure 1A) and its leading compound glycitein, were chemically synthesized in our lab and determined by spectra including 1H-NMR, 13C-NMR, and high resolution mass spectrum (HRMS). The Z-configuration of RY-2f was confirmed by NOESR 1D spectra. The purity of RY-2f and glycitein was analyzed over 98% by HPLC. Genistein and cisplatin were purchased from Sigma-Aldrich (St Louis, MO).

### Cell culture and transfection

Human ovarian cancer cell lines A2780, HEY and OVCA433 were purchased from ATCC. The cisplatin resistant ovarian cancer cell line A2780/CDDP was kindly provided by Prof. Ling-Ya, Pan [56]. Cells were routinely cultured with RPMI-1640 supplemented with 10% FBS, 100 U/mL penicillin and 100 μg/mL streptomycin in a humidified incubator at 37°C in an atmosphere of 5% CO_2_. RPMI-1640 medium and fetal bovine serum (FBS) were purchased from Thermo Scientific. For transfection studies, cells were transiently transfected with myr-AKT1plasmid (constitutive active mutant) or the control vector using Fugene HD (promega). The myr-AKT1 plasmid was constructed as previously reported [57].

### *In vitro* cytotoxicity

The *in vitro* cytotoxicity of RY-2f, glycitein and genistein was measured by MTT (Sigma-Aldrich) assay, as described in the literature [58]. Briefly, 5 × 10^3^ cells per well were plated in 96-well plates and treated with RY-2f, glycitein, genistein or DMSO (diluent) at various concentrations for 48 h. For synergistic effect assay, cells were treated with RY-2f, cisplatin, RY-2f plus cisplatin or DMSO (diluent) for 48 h. Then, the medium with compounds or DMSO was replaced with 180 μL of fresh medium along with 20 μL of MTT solution (MTT dissolved in PBS at 5 mg/mL) in each well and incubated at 37°C for 4 h. Last, the MTT-containing medium was discarded and 150 μL of DMSO per well was added to dissolve the newly formed formazan crystals. Absorbance of each well was determined by a microplate reader (Synergy H4, Bio-Tek) at a 590 nm wavelength. Growth inhibition rates were calculated with the following equation,

$${\text{Inhibition ratio = (OD}}_{\text{DMSO}}{\text{-OD}}_{\text{drug}}{\text{)/(OD}}_{\text{DMSO}}{\text{-OD}}_{\text{blank}}\text{)×100\%.}$$

### Colony formation assay

1 × 10^3^ cells were seeded in six-well plates at a single cell density and treated with RY-2f, cisplatin, RY-2f plus cisplatin, or DMSO (diluent) at various concentrations for 48 h. Then the fresh medium was added to allow cell growth for at least one week. The colonies with more than 50 cells were counted after staining with gentian violet (Solarbio).

### Cell cycle analysis

Cell cycle status was detected by flow cytometry according to a previously published method [59] and analyzed by Multicycle AV (for windows, version 320) software. Briefly, cells were first treated with RY-2f or DMSO at various concentrations for 48 h, and then harvested, washed twice with 1× PBS, and re-suspended in 200 μL of 1× PBS. The cells were fixed in 4 mL of ice-cold 75% ethanol at 4°C overnight and stained with 200 μL of propidium iodide (50 μg/mL, Sigma-Aldrich) and 20 μL of RNase (1 mg/mL, Sigma-Aldrich) to remove RNA in a 37°C water bath for 15 to 20 minutes. The cells were then analyzed by flow cytometry (Cytomics FC 500 MPL, Beckman Coulter). The results were indicated as mean values from three independent determinations.

### Cell apoptosis analysis

To detect apoptosis, cells were incubated with RY-2f or DMSO at different concentrations for 24 h. The cells were harvested, washed twice with cold 1 × PBS, and re-suspended in 200 μL binding buffer at density of 1 × 10^5^ cells / mL. The cells were then stained with 5 μL Annexin-V and PI (BD Biosciences) for 15 min in dark condition at room temperature and subjected to analysis by flow cytometry (Cytomics FC 500 MPL, Beckman Coulter). The early apoptosis was evaluated based on the percentage of cells with Annexin V+/PI−, while the late apoptosis was that of cells with Annexin V+/PI+. The results were indicated as mean values from three independent determinations.

### Western blot analysis

Western blot analysis was performed to determine the expression levels of various proteins in cells. Cells were treated with RY-2f or DMSO at different concentrations for 24 h. Cells were harvested, washed with cold 1 × PBS, and lysed with RIPA lysis buffer (Beyotime) for 30 min on ice, then centrifuged at 12,000 *g* for 15 min at 4°C. The total protein concentration was determined by BCA protein assay kit (Beyotime). Equal amounts (30 μg per load) of protein samples were subjected to SDS-PAGE electrophoresis and transferred on to polyvinylidene fluoride (PVDF) membranes (Millipore). The blots were blocked in 10% non-fat milk, and incubated with primary antibodies, followed by incubation with secondary antibodies conjugated with horseradish peroxidase (HRP). The protein bands were developed with the chemiluminescent reagents (Millipore). Antibodies to p21, Bcl-2, Bad, Bax, cyclin A, cyclin B1, CDK2, pAKT(Ser437), AKT, PI3K (p110 α), mTOR, PTEN were from Santa Cruz Biotechnology. The Antibody to cleaved-PARP-1 was purchased from Cell Signaling Technology. The antibody to β-Actin was purchased from Sigma-Aldrich.

### Small interfering RNA (SiRNA) mediated knockdown of Bax expression

Bax siRNA or Control siRNA were purchase from Genepharma (Shanghai, China) and transfected into cells using Lipofectamine 2000 according to the manufacturer's protocol. The sense and antisense strands of siRNA were beginning at nucleotide 217, 5′ P-UAUGGAGCUGCAGAGGAUGdTdT-3′ (sense) and 5′ P-CAUCCUCUGCAGCUCCAUAdTdT-3′ (antisense) [60]; P represents 5′ phosphate. Total cell lysates were prepared 48 h after transfection to assess the knockdown efficiency by western blot analysis. Otherwise, 24 h after transfection, cells were treated with or without tivantinib for additional 24 h and the cellular apoptosis was evaluated by flow cytometry.

### *In vivo* tumor growth assay

Female BALB/c nude mice at 6–7 weeks of age were purchased from Shanghai Slac Laboratory Animal Co. Ltd. and housed in a specific pathogen free facility. Mice were subcutaneously inoculated with A2780 or A2780/CDDP cells (2 × 10^6^ suspended in 0.2 mL PBS for each mouse). After reaching an average tumor volume of 100 mm^3^, the animals were randomized into groups (*n* = 6) that were treated intraperitoneally with either 30 mg/kg RY-2f (compound dissolved in 0.2 mL olive oil), CDDP (10 mg/kg) or vehicle control (0.2 mL olive oil) thereafter. Administration of vehicle or agents and measurement of tumor growth with a digital caliper were done once every 4 days. Tumor volumes were calculated by the two dimensional sizes of each tumor with the following formula: V = L × W^2^ × 0.52, where V is the volume, L is the length, and W is the width. At the end of experiment, the mice were weighed and sacrificed, and the tumors were weighed and dissected. The animal experimental protocols were approved by the Animal Ethics Committee of Fudan University Shanghai Cancer Center.

### Statistical analysis

The data were calculated using Graph Pad Prism and expressed as mean ± S.E. The values of IC50 were fitted using a nonlinear regression model with a sigmoidal dose response. Comparisons between controls and treated groups were determined by paired t test or one-way ANOVA followed by Tukey's multiple comparison tests. Results were considered statistically significant at the level of *p* < 0.05. Combination index was calculated by CompuSyn software discovered by Chou T.C. et al.

## Abbreviations

PI3K: phosphatidylinositol 3-kinase
PTEN: phosphatase and tensin homolog deleted on chromosome ten
mTOR: the mammalian target of rapamycin
PI: propidium iodide
PARP-1: poly(ADP-ribose) polymerase-1
HPLC: High Performance Liquid Chromatography
MTT: Methyl thiazolyl diphenyl tetrazolium bromide
